# Effects of oleoylethanolamide supplementation on inflammatory biomarkers, oxidative stress and antioxidant parameters of obese patients with NAFLD on a calorie-restricted diet: A randomized controlled trial

**DOI:** 10.3389/fphar.2023.1144550

**Published:** 2023-04-07

**Authors:** Helda Tutunchi, Farideh Zolrahim, Mahlagha Nikbaf-Shandiz, Fatemeh Naeini, Alireza Ostadrahimi, Sina Naghshi, Reza Salek, Farzad Najafipour

**Affiliations:** ^1^ Endocrine Research Center, Tabriz University of Medical Sciences, Tabriz, Iran; ^2^ Student Research Committee, Tabriz University of Medical Sciences, Tabriz, Iran; ^3^ Students’ Scientific Research Center, Tehran University of Medical Sciences, Tehran, Iran; ^4^ Department of Clinical Nutrition, School of Nutritional Sciences and Dietetics, Tehran University of Medical Sciences, Tehran, Iran; ^5^ Nutrition Research Center, Tabriz University of Medical Sciences, Tabriz, Iran

**Keywords:** OEA, inflammation, and NAFLD inflammation, non-alcoholic fatty liver disease, oleoylethanolamide, oxidative stress

## Abstract

**Background:** Oxidative stress is considered a major factor in the pathophysiology of non-alcoholic liver disease (NAFLD). A growing body of evidence indicates that oleoylethanolamide (OEA), a bioactive lipid mediator, has anti-inflammatory and antioxidant properties. This trial investigated the effects of OEA administration on inflammatory markers, oxidative stress and antioxidant parameters of patients with NAFLD.

**Methods:** The present randomized controlled trial was conducted on 60 obese patients with NAFLD. The patients were treated with OEA (250 mg/day) or placebo along with a low-calorie diet for 12 weeks. Inflammatory markers and oxidative stress and antioxidant parameters were evaluated pre-and post-intervention.

**Results:** At the end of the study, neither the between-group changes, nor the within-group differences were significant for serum levels of high-sensitivity C-reactive protein (hs-CRP), interleukin-1 beta (IL-1β), IL-6, IL-10, and tumor necrosis-factor α (TNF-α). Serum levels of total antioxidant capacity (TAC) and superoxide dismutase (SOD) significantly increased and serum concentrations of malondialdehyde (MDA) and oxidized-low density lipoprotein (ox-LDL) significantly decreased in the OEA group compared to placebo at study endpoint (*p* = 0.039, 0.018, 0.003 and 0.001, respectively). Although, no significant between-group alterations were found in glutathione peroxidase and catalase. There were significant correlations between percent of changes in serum oxidative stress and antioxidant parameters with percent of changes in some anthropometric indices in the intervention group.

**Conclusion:** OEA supplementation could improve some oxidative stress/antioxidant biomarkers without any significant effect on inflammation in NAFLD patients. Further clinical trials with longer follow-up periods are demanded to verify profitable effects of OEA in these patients.

**Clinical Trial Registration:** www.irct.ir, Iranian Registry of Clinical Trials IRCT20090609002017N32.

## Introduction

Non-alcoholic fatty liver disease (NAFLD) is recognized as the most frequent chronic liver disease globally (Abd El-Kader and El-Den Ashmawy, 2015). Various estimates of NAFLD prevalence have been reported in the general population (Estes et al., 2020). In 2030, the prevalence of NAFLD is estimated at 33.5% among the adult population and 28.4% among all ages (Estes et al., 2018). The disease spectrum ranges from simple steatosis to non-alcoholic steatohepatitis (NASH), characterized by hepatocyte ballooning and/or lobular inflammation, which may lead to advanced fibrosis, cirrhosis and fatal liver failure (Benedict and Zhang, 2017). Patients with NAFLD, especially those with NASH, often have one or more components of the metabolic syndrome: obesity, dyslipidemia, hypertension, and elevated fasting plasma glucose concentrations or overt type 2 diabetes mellitus (T2DM). Recently, it has been proposed that to rename NAFLD as metabolic dysfunction-associated fatty liver disease (MAFLD) (Tutunchi et al., 2020a; Singh et al., 2021).

Oxidative stress is regarded as the leading cause of various liver diseases (Rolo et al., 2012; Li et al., 2015a). According to the multiple-hit hypothesis, the widely accepted theory in NAFLD pathogenesis, oxidative stress is one of the major mechanisms responsible for the development of liver disorders by stimulating Kupffer cells, hepatic stellate cells, and hepatocytes (Takaki et al., 2013; Ma et al., 2021; Wang et al., 2021). Moreover, accumulating evidence indicates that inflammatory pathways play pivotal roles in the pathogeneses of NAFLD, and continuous inflammation (e.g., pro-inflammatory markers and recruitment of inflammatory cells) stimulates the progression of NASH (Stojsavljević et al., 2014; Kwon et al., 2016). Evidence demonstrates that the severity of NAFLD may be related to increased serum concentrations of inflammatory mediators as well as decreased levels of anti-inflammatory cytokines (Braunersreuther et al., 2012; Paredes-Turrubiarte et al., 2016).

To date, there is no Food and Drug Administration (FDA)-approved pharmacological treatment for NAFLD. Adherence to a calorie-restricted diet and regular physical activity are the backbone of therapy for patients with NAFLD. However, these goals are difficult to implement due to poor adherence. Therefore, adjuvant therapy is proposed as a potential treatment strategy in these patients (Mahjoubin-Tehran et al., 2021). As peroxisome proliferator-activated receptor (PPAR) agonists can improve metabolic dysfunctions, inflammation, and oxidative stress related to liver dysfunctions, agonists of PPARs have gained much attention from the NAFLD and NASH research community (Gross et al., 2017; Fougerat et al., 2020). Oleoylethanolamide (OEA), a potent endogenous PPAR-α agonist, is a bioactive lipid mediator that belongs to the family of endogenous acylethanolamides. OEA acts *via* PPAR-α and has been proposed to exert multiple biological activities such as anti-obesity, anti-inflammatory, and antioxidant effects (Tutunchi et al., 2019; Akbari et al., 2022). OEA administration has been demonstrated to reduce food intake (Fu et al., 2003; Oveisi et al., 2004) and increase across-meal satiety (Fu et al., 2008) through gut-brain communication. Furthermore, OEA induces fatty acid uptake and lipolysis in the adipose tissue and liver and reduces body weight gain in mice and rats (Guzmán et al., 2004; Tutunchi et al., 2019).

The therapeutic effects of OEA in the modulation of various liver disorders have also been reported in several studies. For instance, it has been demonstrated that OEA can decrease lipid synthesis and lipoprotein secretion in the liver (Pan et al., 2018) and improve liver steatosis in rats (Li et al., 2015b) and humans (Tutunchi et al., 2020b; Tutunchi et al., 2020c; Tutunchi et al., 2021). OEA attenuated liver fibrosis through a PPAR-α dependent mechanism (Chen et al., 2015). Misto et al. (2019) have found that OEA enhances fasting-induced liver ketogenesis by activating PPAR-α. Moreover, treatment with OEA significantly alleviated acute liver injury in mice by decreasing plasma alanine aminotransferase (ALT) and aspartate aminotransferase (AST) concentrations, and reducing the histopathological changes. Furthermore, administration of OEA attenuated hepatic apoptosis, inhibited the expression of oxidative stress biomarkers, and elevated the activity of antioxidant enzymes. On the other hand, OEA significantly prevented the expression of pro-inflammatory mediators and suppressed NLRP3 inflammasome in mice (Hu et al., 2020). Therefore, due to the profitable effects of OEA on liver diseases on the one hand and the limitation of clinical trials on antioxidant and anti-inflammatory effects of OEA in NAFLD patients on the other hand, the present trial, for the fist time, assessed the effects of OEA on inflammatory markers, oxidative stress and antioxidant parameters of patients with NAFLD on a calorie-restricted diet.

## Methods

### Trial design and subjects

The methods have been published previously (Tutunchi et al., 2020c; Tutunchi et al., 2021). The present investigation was a randomized, triple-blind, parallel-controlled trial conducted between 2019-February and 2019-July. The design was approved by the ethics committee of Tabriz University of Medical Sciences (IR.TBZMED.REC.1401.425). This trial was registered in the Iranian Registry of Clinical Trials (IRCT20090609002017N32). All of the participants provided written informed consent.

By posting advertisements, subjects willing to participate were recruited from hospitals and clinics in the north-west of Iran. The participants of the current study were obese men and women with NAFLD, who met the following inclusion criteria: age of 20–50 years, body mass index (BMI) of 30–40 kg/m^2^, with a diagnosis of NAFLD (grade I or II) based on ultrasonographic findings.

To assess the severity of fatty liver, ultrasonographic scoring system developed by Hamaguchi *et al* (Hamaguchi et al., 2007) was applied. Hepatorenal echo contrast, bright liver, deep attenuation, and vessel blurring were applied to classify steatosis into three grades including Mild (grade I), Moderate (grade II) and Severe (grade III). As the most severe form of NAFLD patients (grade III) took specific medications for live damage, they were not eligible to enroll to the study (according to defined inclusion criteria) as well as ethical problems.

Exclusion criteria included the use of weight-lowering diets or agents in the last 6 months, pregnancy, lactation, menopause, smoking, being professional athlete, addiction to alcohol (>10 g/d for women and >20 g/d for men), any evidence of chronic infections, diabetes, or autoimmune diseases, high blood pressure, kidney or thyroid dysfunction, polycystic ovary syndrome, cardiovascular, gastrointestinal, cancer, or hepatocellular diseases, the use of anti-diabetic, antioxidant, or multivitamin-mineral supplements in the last 3 months, the use of any medication or dietary supplements that affect glucose and lipids metabolism during the last 3 months or attending another clinical trial. In addition, the individual characteristics and medical history of each patient were collected.

### Sample size

The sample size was determined according to mean and standard deviation (SD) of TNF-α on the basis of previous trial by Payahoo *et al.* (Payahoo et al., 2018). We calculated that a sample size of 30 subjects was required for each group of the trial considering a power of 80% and 95% significance level and an drop-out rate of 20%.

### Randomization and intervention

Using the block randomization method in blocks stratified by sex and grading of NAFLD, the participants were randomly allocated to either the intervention group (*n* = 30) or the control group (*n* = 30) for 12 weeks. Until the end of the analysis, patients and researchers as well as statistical consultant were blind to the group assignments of the trial. Synthesis of OEA was conducted at Nutrition Research Center of Tabriz University of Medical Sciences and has been described in detail in previous clinical trial (Laleh et al., 2018; Tutunchi et al., 2020c). The daily OEA dose received by each patient was considered at 250 mg based on the previous study on obese patients (Laleh et al., 2018). The intervention group was treated with OEA (125 mg), twice a day, half an hour before lunch and dinner meal, and the placebo group received two capsules of 125 mg starch for 12 weeks. The capsules of both groups (in the same shape, size, and color) were delivered to patients monthly.

Individual calorie-restricted diet (−500 kcal of total energy expenditure) was designed for participants of each group. Macronutrient distribution range for fat, protein, and carbohydrates was respectively set at 30%, 15%, and 55% of daily total energy intake.

To increase compliance, all patients received brief daily cell phone reminders to take the supplements and emphasis physical activity and weight-loss dietary plans given to them. Besides, every 2 weeks, participants were returned to clinic to assess diet based on data obtained from 3- day food records. Participants were also directly asked for adverse events. Assessment of supplement adherence was conducted using unused capsule counts of each patient (patients consuming >90% of the capsules delivered during the study were considered as high adherence to the study intervention). Data on calorie intake, obtained from the 3-day food record, was applied for assessing adherence to the dietary plans given to the participants.

### Dietary intake and physical activity assessment

Dietary intake information was collected based on 3-day food record (two non-consecutive days and one weekend day per individual) and analyzed by the Nutritionist IV software (First Databank, San Bruno, CA, United States) at the onset and end of the study. International Physical Activity Questionnaire-Short Form (IPAQ-SF) was also applied to evaluate physical activity, based on metabolic equivalent task minutes per week (MET, min/week) (Craig et al., 2003).

### Anthropometric and biochemical measurements

Fasting body weight (kilograms) and height were assessed on a calibrated Seca balance beam scale with height rod while patients were barefoot and wearing light clothe. Other anthropometric indices, including waist circumference (WC) and hip circumference (HC) were evaluated using the standard methods. BMI, waist-to-hip ratio (WHR) and waist-to-height ratio (WHtR) were also computed.

All the biochemical measurements were performed at pre-and post-intervention. A 5 ml venous blood sample was collected from each patient after a 12-h overnight fasting and frozen at −80°C immediately after collection. Serum concentration of high-sensitivity C-reactive protein (hs-CRP) was assessed using the immunoturbidimetry method (Pars Azmoon Co., Tehran, Iran). Serum levels of interleukin-1 beta (IL-1β), IL-6, IL-10, tumor necrosis-factor α (TNF-α) were measured by commercial enzyme-linked immunosorbent assay (ELISA) kits (Bioassay Technology Laboratory, Shanghai City, China). Total antioxidant capacity (TAC) was assessed through the colorimetric method by Randox total antioxidant status kit (Randox Laboratories, United Kingdom). The levels of glutathione peroxidase (GSH-Px) and superoxide dismutase (SOD) were evaluated by spectrophotometric method using Ransel and Ransod kits, respectively (Randox Laboratories, United Kingdom). The spectrophotometric thiobarbituric acid reactive substances (TBARS) method was used to determine serum level of malondialdehyde (MDA). The Aebi’s method was used for measuring catalase activity. Serum oxidized-low density lipoprotein (ox-LDL) was detected by ELISA (Mercodia AB, Uppsala, Sweden), according to manufacturers’ specifications. Serum concentrations of total cholesterol (TC), high-density lipoprotein cholesterol (HDL-C), low-density lipoprotein cholesterol (LDL-C) and triglyceride were determined by commercial kits (Pars-Azmoon Co., Tehran, Iran).

### Statistical analysis

An intention-to-treat approach was used in all analyses. The Shapiro-Wilk test and histograms were applied to check the normality of variables. Continuous variables with normal distribution were reported as mean (SD) and non-normal variables were presented as median (25th and 75th percentiles). The qualitative variables were represented with frequency (percentage). Comparison of baseline characteristics of patients between study groups was performed using the independent samples *t*-test or Mann–Whitney *U* test. To test within-group differences, Paired samples *t*-test or Wilcoxon signed-rank test were applied. Fisher’s exact test run for analyzing between-group changes of qualitative data. To compare the two groups at the end of the trial adjusting for baseline values and potential confounders (i.e., age, alterations in physical activity, energy intake, and BMI), analysis of covariance (ANCOVA) was run. The correlation of serum oxidative stress and antioxidant parameters with anthropometric indices and metabolic parameters was analyzed using Pearson’s correlation coefficient test. Two-tailed *p*-value <0.05 was regarded to be statistically significant. All statistical analyses were done using SPSS, version 23.0 (SPSS Inc., Chicago, IL, United States).

## Results

**FIGURE 1:**
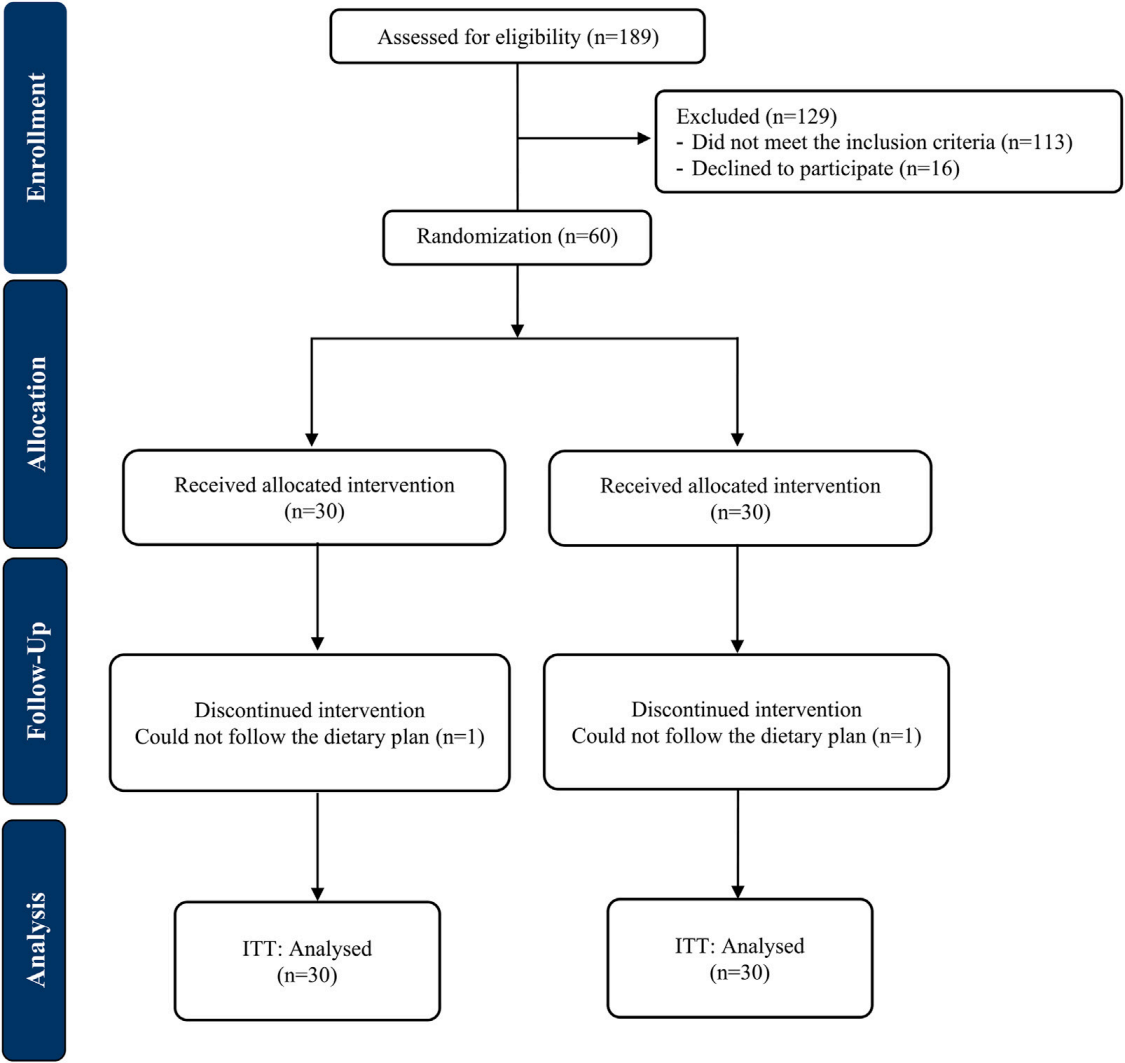
Study flow diagram (ITT, Intention to treat).

The CONSORT flowchart of the trial is exhibited in Figure 1. A total of 58 participants completed the trial; one patient in each arm was lost to follow-up due to non-adherence to dietary recommendations. No side effects were reported by those who ended the trial. There were no significant alterations between the study groups in the baseline characteristics of patients including demographic characteristics, physical activity levels and the severity of fatty liver (Table 1).

**TABLE 1:** Baseline characteristics of the study participants.

	OEA (*n* = 30)	Placebo (*n* = 30)	*p*
Age (years)	41.78 (9.77)	42.39 (11.77)	0.839^a^
Gender, n (%)			1.00^b^
Males	14 (46.66)	14 (46.66)	
Females	16 (53.34)	16 (53.34)	
Education, n (%)			0.403^b^
Illiterate	0 (0)	1 (3.33)	
Diploma and lower	23 (76.66)	22 (73.33)	
Bachelors and higher	7 (23.34)	7 (23.34)	
Employment, n (%)			
Employed	19 (63.34)	17 (56.67)	0.207^b^
Unemployed	11 (36.66)	13 (43.33)	
Marital status, n (%)			0.369^b^
Single	3 (10.00)	5 (16.67)	
Married	27 (90.00)	25 (83.33)	
Physical activity level, n (%)			
Very low	12 (40.00)	13 (43.33)	0.419^b^
Low	11 (36.66)	9 (30.00)	
Moderate and high	7 (23.34)	8 (26.67)	
Severity of fatty liver, n (%)			1.00^b^
Mild	19 (63.33)	19 (63.33)	
Moderate	11 (36.67)	11 (36.67)	
Severe	0 (0)	0 (0)	

BMI, body mass index.; OEA, oleoylethanolamide.

Age is presented as mean (SD); other variables are presented as number (%).

^a^ Independent samples *t*-test.

^b^ Fisher’s exact test.

At baseline, there were no significant differences between the two groups, for anthropometric indices, dietary intakes and physical activity (Table 2). Physical activity of the participants did not change significantly throughout the trial (*p* = 0.206; data not shown).

**TABLE 2:** Anthropometric indices, dietary intake and physical activity of the patients at baseline.

	OEA (*n* = 30)	Placebo (*n* = 30)	*p*
Weight (kg)	87.32 (13.79)	85.93 (14.77)	0.720^a^
BMI (kg/m^2^)	33.79 (6.69)	35.53 (7.12)	0.335^a^
Energy (kcal)	2351.17 (514.61)	2287.39 (429.11)	0.614^a^
Carbohydrate (g)	313.58 (98.75)	327.12 (111.51)	0.632^a^
Protein (g)	72.14 (28.17)	69.91 (24.57)	0.756^a^
Total fat (g)	91.28 (36.79)	87.33 (34.12)	0.680^a^
Cholesterol (mg)	153.77 (97.14, 391.67)	139.67 (94.33, 318.77)	0.501^b^
SFA (g)	13.46 (7.79, 19.65)	14.70 (9.65, 18.49)	0.389^b^
MUFA (g)	14.95 (6.13)	16.11 (7.03)	0.505^a^
PUFA (g)	27.66 (16.95, 39.55)	23.91 (10.16, 31.33)	0.485^b^
Dietary fiber (g)	15.15 (7.41, 19.74)	17.79 (5.51, 21.66)	0.335^b^
Physical activity (METs)	887.78 (456.50, 1953.33)	845.11 (234.19, 1789.11)	0.715^b^

BMI, body mass index; MD, METs, metabolic equivalents (MET-minutes/week); MUFA, monounsaturated fatty acids; OEA, oleoylethanolamide; PUFA, poly unsaturated fatty acids; SFA, saturated fatty acids. Mean (standard deviation) are presented for normally distributed data. Median (25th and 75th percentiles) are presented for data not normally distributed.

^a^ *p*-value based on Independent sample *t*-test.

^b^ *p*-value based on Mann-Whitney *U* test.

*p* < 0.05 is statistically significant.

As presented in Table 3, there were no significant differences in inflammatory biomarkers as well as oxidative stress/antioxidant parameters between study groups at baseline. In terms of inflammatory markers, hs-CRP decreased significantly in both study groups, no significant between-group alteration was observed for this parameter at the end of the study, though. Neither the between-group changes, nor the within-group differences reached statistical significance for other markers of inflammation including IL-1β, IL-6, IL-10, and TNF-α. Serum levels of TAC and SOD significantly increased in both groups. Greater increase in TAC and SOD levels was found in the OEA group compared to the control at endpoint of the study (*p* = 0.039 and 0.018, respectively) after adjusting for potential confounders including baseline values, age, changes in physical activity, energy intake, and BMI. Moreover, serum MDA and ox-LDL concentrations decreased in the OEA group compared to the control at study endpoint (*p* = 0.003 and 0.001, respectively). However, between-group alterations adjusted for above-mentioned confounding variables revealed no meaningful differences between the study groups in terms of GSH-Px and catalase.

**TABLE 3:** Inflammatory biomarkers and oxidative stress/antioxidant parameters of the patients throughout study.

	OEA (*n* = 30)	Placebo (*n* = 30)	MD	95% CI	*p*
IL-6 (pg/mL)					
Baseline	18.67 (6.76)	21.68 (8.78)	−3.01	−7.11, 1.10	0.138^c^
End	16.98 (5.56)	20.17 (6.56)	−5.19	−11.97, 1.53	0.125^d^
MD	−1.69	−1.50			
95% CI	- 4.49, 1.11	−4.49, 1.48			
*p* ^a^	0.236	0.324			
IL-10 (ng/ml)					
Baseline	118.33 (74.37, 165.23)	129.78 (90.51, 173.98)	−11.45	−39.84, 16.93	0.429^e^
End	124.59 (89.44, 173.89)	134.48 (84.39, 177.78)	−13.57	−55.71, 29.89	0.529^d^
MD	6.26	4.70			
*p* ^b^	0.193	0.234			
IL-1β (pg/mL)					
Baeline	13.23 (4.49)	15.11 (6.67)	−1.88	−4.88, 1.11	0.205^c^
End	11.78 (3.39)	14.82 (5.59)	−4.78	−14.69, 6.09	0.101^d^
MD	−1.45	−0.29			
95% CI	−3.76, 0.86	−1.78, 1.18			
*p* ^a^	0.218	0.701			
TNF-α (ng/L)					
Baseline	53.71 (40.37, 157.23)	50.12 (38.22, 111.56)	3.59	−23.93, 31.10	0.798^e^
End	51.97 (36.37, 145.23)	49.36 (40.23, 127.87)	4.55	−12.81, 20.38	0.591^d^
MD	−1.74	−0.76			
*p* ^b^	0.409	0.609			
hs-CRP (mg/L)					
Baseline	7.14 (4.08, 17.33)	5.78 (3.39, 12.98)	1.36	−1.00, 3.72	0.256^e^
End	4.56 (3.22, 21.11)	3.33 (2.13, 19.67)	2.49	−3.11, 7.96	0.311^d^
MD	−2.58	−2.45			
*p* ^b^	**0.038**	**0.013**			
TAC (mmol/L)					
Baseline	0.81 (0.07)	0.75 (0.17)	0.06	−0.008, 0.129	0.075^c^
End	1.33 (0.15)	1.11 (0.19)	0.22	0.02, 0.59	**0.039 ** ^**d**^
MD	0.52	0.36			
95% CI	0.16, 0.88	0.03, 0.70			
*p* ^a^	**0.005**	**0.032**			
GSH-Px (U/g Hb)					
Baseline	29.86 (6.89)	31.07 (3.87)	−1.21	−4.15, 1.72	0.411^c^
End	34.48 (9.16)	33.59 (7.71)	1.59	−0.87, 3.94	0.173^d^
MD	4.62	1.89			
95% CI	1.68, 7.56	−0.24, 4.01			
*p* ^a^	**<0.001**	0.081			
Catalase (U/g Hb)					
Baseline	58.78 (11.77)	61.55 (19.11)	−2.77	−11.05, 5.50	0.511^c^
End	60.05 (19.22)	61.12 (21.09)	−2.89	−7.63, 3.83	0.412^d^
MD	1.27	−0.43			
95% CI	−1.62, 4.16	−2.13, 1.26			
*p* ^a^	0.387	0.621			
SOD (U/mg Hb)					
Baseline	1693.12 (105.77)	1733.11 (129.53)	−39.99	−87.84, 7.87	0.095^c^
End	1933.13 (121.55)	1823.97 (152.31)	112.16	29.76, 207.09	**0.018 ** ^**d**^
MD	240.02	90.86			
95% CI	87.80, 392.3	20.83, 160.90			
*p* ^a^	**<0.001**	**0.011**			
MDA (nmol/mL)					
Baseline	4.08 (0.55)	3.88 (0.33)	0.20	−0.03, 0.43	0.088^c^
End	2.33 (0.97)	3.33 (0.98)	−1.78	−0.73, −2.87	**0.003** ^d^
MD	−1.75	−0.55			
95% CI	−2.79, −0.71	−1.22, 0.12			
*p* ^a^	**0.001**	0.109			
Ox-LDL (U/L)					
Baseline	76.21 (14.28)	74.88 (21.87)	1.33	−8.29, 10.95	0.795^c^
End	71.55 (17.77)	72.51 (24.61)	- 2.39	−6.12, −1.77	**0.001** ^d^
MD	−4.66	−2.37			
95% CI	−8.52, −0.81	−6.28, 1.53			
*p* ^a^	**0.018**	0.234			

GSH-Px, glutathione peroxidase; hs-CRP, high-sensitivity C-reactive protein; IL, interleukin; MD, Mean/Median of difference; MDA, malondialdehyde; OEA, oleoylethanolamide; ox-LDL, oxidized-low density lipoprotein; SOD, superoxide dismutase; TAC; total antioxidant capacity; TNF-α, tumor necrosis factor-α. Mean (standard deviation) are presented for normally distributed data. Median (25th and 75th percentiles) are presented for data not normally distributed.

^a^ *p*-value based on Paired sample *t*-test.

^b^ *p*-value based on Wilcoxon signed-rank test.

^c^ *p*-value based on Independent sample *t*-test.

^d^ *p*-value based on ANCOVA, adjusted for baseline values, age, changes in physical activity, energy intake, and BMI.

^e^ *p*-value based on Mann-Whitney *U* test.

*p* < 0.05 is statistically significant.

Bold values indicates statistically significant differences (*p* < 0.05).

Correlation coefficients of serum oxidative stress and antioxidant parameters with anthropometric indices and metabolic parameters are presented in Table 4. A significant inverse relationship was detected between percent of changes in TAC with percentage of changes in BMI (*p* = 0.016) and WHR (*p* = 0.027) as well as an inverse relationship between percent of changes in serum SOD level with percent of changes in BMI (*p* = 0.018) and WC (*p* = 0.001) in the intervention group. However, we found a positive correlation between percent of changes in MDA with percent of changes in BMI (*p* = 0.022) and WHR (*p* = 0.041) as well as a positive relationship between percent of changes in serum ox-LDL level with percent of changes in BMI (*p* = 0.012) and WC (*p* = 0.001) in the intervention group. No meaningful relationships were detected between serum oxidative stress and antioxidant parameters with other anthropometric indices as well as metabolic parameters in the study groups.

**TABLE 4:** Correlation coefficients among serum oxidative stress and antioxidant parameters and anthropometric indices and metabolic parameters.

Percnt of changes	OEA (*n* = 30)						Placebo (*n* = 30)					
	Percent of changes						Percent of changes					
	TAC	GSH-Px	Catalase	SOD	MDA	Ox-LDL	TAC	GSH-Px	Catalase	SOD	MDA	Ox-LDL
**BMI**	−0.461* (**0.016**)	−0.667 (0.301)	−0.207 (0.419)	−0.470 * (**0.018**)	0.409 * (**0.022**)	0.509 * (**0.012**)	−0.238 (0.173)	−0.207 (0.601)	0.051 (0.859)	−0.289 (0.136)	0.416 (0.495)	0.116 (0.087)
**WC**	−0.361 (0.259)	−0.055 (0.487)	−0.177 (0.591)	−0.365* (**0.001**)	0.154 (0.355)	0.440 * (**0.001**)	−0.641 (0.559)	−0.104 (0.314)	−0.105 (0.750)	−0.440 (0.316)	0.091 (0.852)	0.204 (0.227)
**HC**	−0.062 (0.800)	−0.078 (0.706)	−0.598 (0.789)	−0.131 (0.801)	0.339 (0.840)	0.009 (0.678)	−0.026 (0.771)	−0.015 (0.837)	0.027 (0.881)	−0.405 (0.809)	0.038 (0.825)	0.049 (0.621)
**WHR**	−0.377* (**0.027**)	−0.315 (0.153)	−0.308 (0.079)	−0.409 (0.088)	0.431* (**0.041**)	0.119 (0.227)	−0.379 (0.163)	−0.155 (0.801)	−0.101 (0.851)	−0.407 (0.097)	0.639 (0.208)	0.029 (0.101)
**WHtR**	−0.131 (0.725)	−0.139 (0.222)	−0.087 (0.709)	−0.158 (0.443)	0.633 (0.199)	0.117 (0.403)	−0.405 (0.613)	−0.253 (0.852)	0.133 (0.391)	−0.088 (0.629)	0.077 (0.733)	0.331 (0.737)
**TC**	−0.101 (0.469)	−0.421 (0.651)	−0.071 (0.843)	−0.293 (0.669)	0.177 (0.809)	−0.131 (0.725)	−0.097 (0.234)	−0.022 (0.605)	0.130 (0.933)	−0.081 (0.409)	0.182 (0.429)	0.431 (0.109)
**TG**	−0.014 (0.133)	−0.310 (0.411)	−0.051 (0.401)	−0.404 (0.087)	0.038 (0.123)	−0.131 (0.725)	−0.335 (0.093)	−0.333 (0.120)	−0.444 (0.601)	−0.199 (0.173)	0.051 (0.733)	0.531 (0.144)
**LDL-C**	−0.279 (0.320)	−0.033 (0.197)	−0.126 (0.805	−0.555 (0.879)	0.731 (0.877)	−0.131 (0.725)	−0.468 (0.701)	−0.403 (0.626)	−0.539 (0.727)	−0.011 (0.311)	0.008 (0.221)	0.491 (0.085)
**HDL-C**	0.045 (0.103)	0.159 (0.091)	0.009 (0.558)	0.058 (0.135)	0.108 (0.933)	−0.131 (0.725)	0.188 (0.249)	−0.039 (0.885)	0.008 (0.885)	0.181 (0.093)	−0.338 (0.097)	−0.229 (0.168)

BMI, body mass index; GSH-Px; glutathione peroxidase; HC, hip circumference; HDL-C, high-density lipoprotein cholesterol; LDL-C, low-density lipoprotein cholesterol; MDA, malondialdehyde; OEA, oleoylethanolamide; ox-LDL, oxidized-low density lipoprotein; SOD, superoxide dismutase; TAC, total antioxidant capacity; TC, total cholesterol; TG, triglyceride; WC, waist circumference; WHR, waist to hip ratio; WHtR, waist to height ratio. *r(*p*) based on the Pearson’s correlation coefficient test.

*p* < 0.05 is statistically significant.

## Discussion

Little has been investigated about the effects of OEA on inflammation and oxidative stress, main pathological factors contributing to the initiation and progression of various liver diseases, in patients with NAFLD. To our knowledge, the present clinical trial is the first study to assess the effects of OEA supplementation on inflammatory indices, oxidative stress and antioxidant parameters in patients with NAFLD. Based on the obtained results, after adjusting for possible confounders, OEA supplementation caused a remarkable increase in TAC and SOD levels in obese patients with NAFLD on a calorie-restricted diet. However, treatment with OEA did not have a significant effect on inflammatory biomarkers including hs-CRP, IL-1β, IL-6, IL-10, and TNF-α. Furthermore, a significant decrease in serum MDA and ox-LDL concentrations were found by OEA supplementation. We also found significant correlations between percentage of changes in serum oxidative stress and antioxidant parameters with percentage of changes in some anthropometric indices in the intervention group.

In contrast with aforementioned results, Payahoo *et al* (Payahoo et al., 2018) concluded that OEA supplementation (250 mg for 8 weeks) could significantly reduce serum concentrations of IL-6 and TNF-α in healthy obese people. Moreover, a significant reduction in the expression levels of nuclear factor-kappa B (NF-kB) and IL-6 was observed in obese patients with NAFLD after OEA supplementation (Tutunchi et al., 2021). On the other hand, in the recent investigation by Sabahi et al. (2022), OEA treatment (300 mg/day) could improve the short-term inflammatory and oxidative stress status in patients with acute ischemic stroke. A significant reduction in serum levels of IL-1β, monocyte chemoattractant protein-1 (MCP-1), and TNF-α were also found by OEA supplementation (5 mg/kg) in alcohol intoxicated rats (Antón et al., 2017). In addition, an animal study conducted by Sun et al. (2007) demonstrated that administration of 10 mg/kg OEA could significantly prevent the expression of cyclooxygenase-2 (COX-2) in wild-type mice. Anti-inflammatory characteristics of OEA have been attributed to the PPAR-α activation (Yang et al., 2016). OEA as a potent endogenous PPAR-α agonist exerts anti-inflammatory effects through increasing the expression of IkB as NF-kB inhibitory protein, inhibiting lipopolysaccharide (LPS)-induced NF-kB activation, suppressing the expression of COX-2 as a NF-kB regulated protein, reducing the expression of vascular cell adhesion molecule-1 (VCAM-1) and intracellular adhesion molecule 1 (ICAM-1) as molecules involved in the inflammatory response, interfering with the extracellular-signal-regulated kinase (ERK)1/2-dependent signaling cascade, and attenuating the expression of genes coding for inflammatory biomarkers including IL-6, IL-1β, and TNF-α (Yang et al., 2016; Payahoo et al., 2018; Tutunchi et al., 2021). On the other hand, as OEA is an endogenous ligand for hypoxia-inducible factor 1 (HIF-α), it might alleviate inflammation (Diao et al., 2022). HIF-1α inhibition could be a potential strategy for limiting exacerbated inflammatory responses because it caused sustained inflammation by activating NF-kB pathway (Palazon et al., 2014). It is important to note that discrepancies between our conclusions and above-mentioned findings might be explained by different nature and design of the investigations as well as supplementation with various dosages of OEA for dissimilar periods of time. In addition, different methods and various ELISA kits to measure serum concentrations of inflammatory biomarkers may account for the apparent discordant results and conclusions. Moreover, follow-up period may not be sufficient enough to affect serum inflammatory biomarkers in the present study.

In line with our results, a recent animal study by Giudetti et al. (2021) revealed a meaningful reduction in the amounts of MDA and carbonylated proteins after OEA treatment. In the same study, OEA significantly increased the activity of antioxidant enzymes including SOD, catalase, and GSH-Px (Giudetti et al., 2021). Additionally, oral administration of 10 mg/kg/day OEA for 8 weeks attenuated oxidative stress, endothelial cell damage, and early atherosclerotic plaque formation in atherosclerotic mice (Ma et al., 2015). Moreover, in plasma samples of healthy subjects, copper-induced lipid peroxidation was reduced by OEA incubation (1 µM for 7 h) (Zolese et al., 2008). The suspected mechanisms responsible for the effects of OEA on oxidative stress and antioxidant parameters are not yet fully understood. OEA acts as a potent antioxidant through enhancing PPAR-α signaling, inhibiting LPS-induced oxidative/nitrosative stress, decreasing the expression of apoptosis-related proteins, preventing lipid peroxidation, attenuating the expression of nuclear factor erythroid 2-related factor 2 (Nrf-2) as a key transcription factor involved in inflammation and antioxidant responses, and reducing the production of reactive oxygen species (ROS) (Fan et al., 2014; Sayd et al., 2014; Payahoo et al., 2018; Giudetti et al., 2021). We found significant correlation between the percentage of changes in serum oxidative stress and antioxidant parameters and the percentage of changes in some anthropometric indices in the OEA group. In the interpretation of these findings, it should be considered that improvement in oxidative stress and antioxidant factors by OEA supplementation might be attributed to improvement in anthropometric parameters. It is documented that obesity can induce systemic oxidative stress (Manna and Jain, 2015). OEA could improve obesity by regulating energy hemostasis, modulating lipid and glucose metabolism, stimulating the expression of genes associated with fatty acid oxidation and lipolysis, and controlling appetite and food intake through induction of satiety-related genes expression (Laleh et al., 2018). Therefore, improvement in anthropometric parameters by OEA supplementation may contribute to the reduction of oxidative stress through lowering tissue lipid levels and inflammation, alleviating endothelial dysfunction and impaired mitochondrial function, decreasing hyperglycemia, and improving hyperleptinemia (Manna and Jain, 2015).

### Strengths and limitations

The inclusion of patients newly diagnosed with NAFLD, perfect adherence to the intervention, using a specified weight-loss diet for all participants, frequent contact with the patients through visit sessions and telephone, robust statistical analysis and consideration of probable confounders were some of the strength points of present trial. However, there are some weaknesses to be considered for data interpretation. First, serum concentration of OEA, the most appropriate method for evaluating the adherence of participants to the intervention, was not measured due to funding restrictions. Second, self-reporting of dietary intake and physical activity may lead to the low or high reporting bias. Third, although ultrasonography is the preferred initial non-invasive and relatively low-cost test for diagnosing NAFLD, it has been shown to be less accurate in diagnosis of mild steatosis. Thus, some cases of mild steatosis might have been missed in this clinical trial. Finally, as it is documented that the biological effects of OEA are mainly mediated through PPAR‐α signaling pathway, it was better to measure serum level of PPAR‐α. However, funding constraints did not allow for measuring serum concentration of PPAR‐α in our patients.

## Conclusion

In summary, OEA supplementation (250 mg for 12 weeks) significantly improved oxidative stress and antioxidant parameters of obese patients with NAFLD on a calorie-restricted diet, while no signicant changes were found in terms of inflammatory biomarkers. Although obtained results indicate that OEA might be a promising therapy option for obese patients with NAFLD, further high-quality randomized clinical trials with longer follow-up periods are demanded to verify profitable effects of OEA in these patients.

## Data Availability

The raw data supporting the conclusion of this article will be made available by the authors, without undue reservation.
